# Nurses' Recreations

**Published:** 1887-03-05

**Authors:** 


### NURSES' RECREATIONS.

"All work and no play makes Jack a dull boy"—in these times, a *very* dull boy. Still more is this the case with Jean, who may be considered a feminine Jack. Now that nursing is a recognised profession, whose members are numbered by thousands, it has a claim to be regarded from every point of view. Too often it has been, and we fear still is, the habit of people to think only of what can be got out of nurses. They have been thought of, no doubt, as ministering angels in times of sickness; but still as angels who have few or no wants of their own;—who are able and willing to give all, asking for nothing again. That is not a practical way of looking at the matter. It does not pay the public, and it breaks down and kills the nurses. The ministering angel theory is very sweet and agreeable; perhaps as much so to nurses as to patients. But it is a theory only, and, unfortunately, does not work well. Nurses are very human, and have stomachs and livers, brains and nervous systems. No creatures so endowed can toil continuously without injury. With them, as with other human beings, work must alternate with play; labour with leisure and relaxation.

There is a special need for recreation in the case of sick-nurses, because of the peculiar nature of their occupation. Their calling makes the greatest possible demands on them, alike on brain and heart, on mind and sympathy. A conscientious nurse—and most nurses are conscientious—gives herself up entirely to the work of the moment. That work is unceasing. The patient is always there, always needing some thought or service, always keeping the mind anxious and the hands busy. The range of duty, too, is narrow and limited. The repetition of like offices hour after hour, day after day, week after week; the passive endurance of the same monotonous round; the same querulousness on the part of the patient; the same disregard on the part of friends—all these constitute a series of duties, a painfulness of service, such as no one without experience can comprehend or even imagine.

Under such circumstances, one of two things will inevitably happen. Either leisure and recreation must be obtained, or the nurse, sooner or later, breaks down. This is as certain as the simplest sum in arithmetic. The human machine is constructed to "go" for a certain period under certain conditions. Alter the conditions, and the machine will not "go." Nothing can make it. No necessity of circumstance, no nobility of duty, no elevation of motive, no energy of enthusiasm, can unmake or modify a single physiological fact. Grant suitable conditions, and the machine will continue to work for its destined period. Deny the conditions, and it will be broken up and become useless. It is a pity these physiological certainties are not believed in. A deep-rooted scientific scepticism is widely prevalent, and especially among women. The educated ladies, whose hearts are warm towards hospitals (a few of whom become nurses and matrons), have seldom any more real faith in the elementary principles of physiology, any more working grasp of those principles, than the less educated nurses whom they employ. That is a real and serious evil. There is a want of "grip" in the feminine mind for general principles, if they appear to be in the least degree of an abstract character. To an observant eye, the facts of physiology are concrete enough, but the processes are sometimes slow, and being slow, the results are seldom realised until much serious mischief is brought about.

What is to be done? The first thing to do is to persuade matrons of hospitals and heads of families who employ nurses, that there really is danger ahead. That is perhaps not so very difficult in the case of the hospital matrons, many of whom are decidedly above the average of their sex in point of reasonableness and good-sense. But when we come to consider the case of the sick-nurse in the family, we have much less hope of establishing any intelligent convictions in the mind. The mental habits of the average Englishwoman of the wealthier class are terribly conventional, not to say priggish. A new idea, be it never so small, never so obviously sound, never so "proper," is still "new," and therefore to be suspected, and most likely severely reprobated, solely on the ground of its unfamiliarity. The ordinary woman seems to be absolutely destitute of originality and intellectual courage. According to her, "that which has been," is not only "that which *shall be*," but also that which *ought to be*. With many women argument is vain and reasoning ridiculous.

But supposing the difficulty of convincing the employers of nurses to be overcome, what recreations are available? Theatres are expensive, and not very amusing. Besides, the class of innocent pleasures most suitable for nurses are those which give a good measure of physical exercise. Singing, music, and dancing are as old as the world, and highly conducive to health. The difficulty is to find opportunities for their exercise. If the suggestion made by Miss Manson and others, that nursing institutions for private patients be established in connection with the general hospitals, were carried out, all kinds of pleasant and healthy amusements might at once be set on foot. Then, by a careful regulation of the terms of outside work, and an orderly system of periodical return to the central institution, each nurse might secure a fair share of recreation and rest, with a corresponding elasticity of health and fitness for professional duty.

A carefully considered series of gymnastic exercises adapted to young women would be invaluable. Each central nursing institution should have a thoroughly equipped female gymnasium, under the authority of a competent teacher. If exercises of this kind were regular and systematic, the nursing profession would be robbed of many of its disabilities and hardships, and its members would probably begin to take high rank among healthy and vigorous women. It cannot be too much insisted upon that nurses are instruments adapted to the production of certain results. The greater the initial and permanent perfection of the instrument, the better will be the result. That is looking at the matter from a purely economic point of view. We must remember, however, that we are dealing with *human* instruments; and therefore we must not only consider the economic aspect of the question, but also the sentimental and the moral. The public will be wise, for its own sake, to train a skilful body of sick-nurses, and to protect their health with a view to their efficiency. But we desire to see everyone taking due thought for them on the higher grounds of human regard and religious duty. No better and simpler position can be taken than this: "Whatsoever ye would that men should do unto you, do ye even so unto them." If this elementary, but profound, principle were universally applied, nurses would have no just ground for complaint. The subject is admittedly beset with difficulties, but that is no reason whatever why its consideration should be denied or even delayed. Admit that the general principles here set forth are in the main sound, and it is *ipso facto* admitted that their realisation in practice should be immediately and energetically sought. We commend the question to all who are connected with the nursing profession, or who have the interests of nurses at heart.

